# Supplementary material for: Mrg15 stimulates Ash1 H3K36 methyltransferase activity and facilitates Ash1 Trithorax group protein function in Drosophila
Source: Nat Commun. 2017 Nov 21;8:1649. doi: 10.1038/s41467-017-01897-3 (PMC5696344; doi:10.1038/s41467-017-01897-3)
Supplement: Supplementary file 1 — Supplementary Information [file 41467_2017_1897_MOESM1_ESM.pdf]

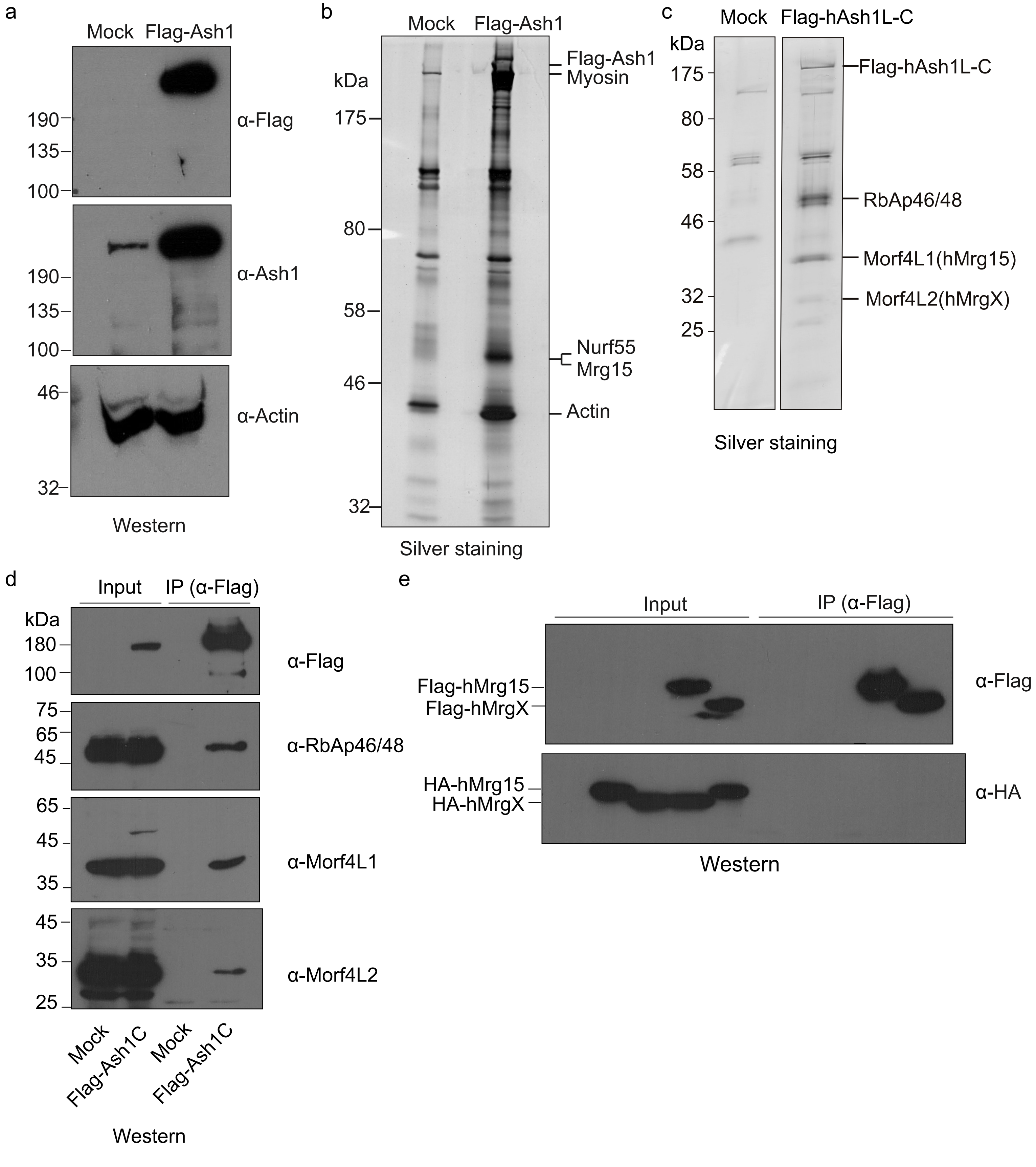

Supplementary Figure 1. Ash1 forms a stable complex with Mrg15 and Nurf55. (a) Western blot results using antibodies against Flag, Ash1 and Actin to show the expression level of Ash1 in mock or Flag-Ash1 S2 cells. (b) Silver staining result of affinity purified materials from S2 cells expressing mock- or full length Flag-Ash1. *: common contaminant proteins. (c) Silver staining result of affinity purified materials from HEK293f cells expressing mock or hFlag-Ash1L-C. (d) Western blot results using antibodies against Flag, RbAp46/48, MORF4L1 and MORF4L2 to confirm the mass spectrometry results of affinity purification.(e) Co-IP results show that hMrg15 and hMrgX exist in a mutually exclusive manner. Combinations of Flag- and HA-tagged hMrg15 and / or hMrgX expression vectors were co-transfected into HEK293F cells and then Co-IP experiments were performed with nuclear extracts prepared from these cells.

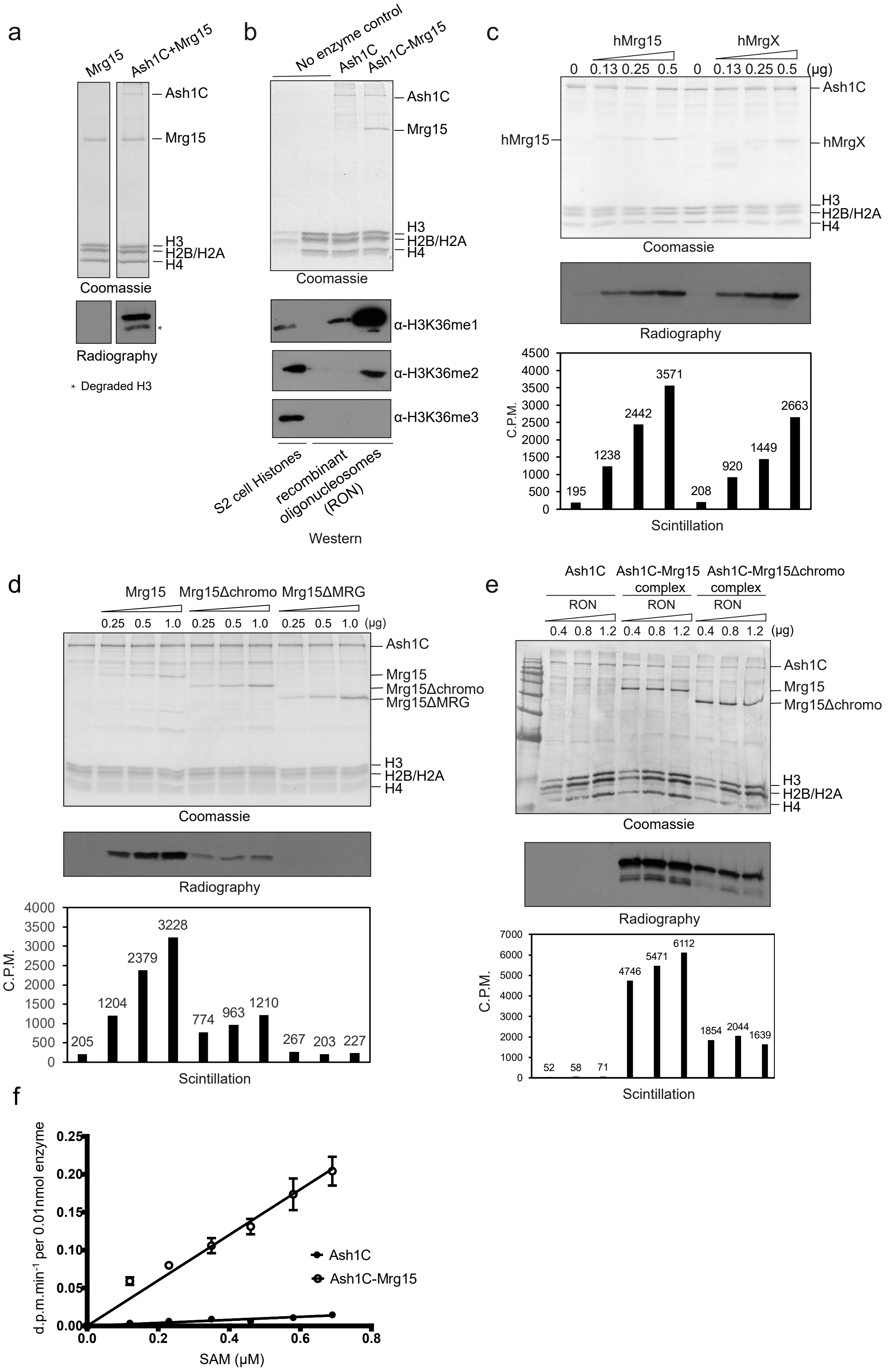

Supplementary Figure 2. Mrg15 stimulates the histone methyltransferase activity of Ash1C in vitro. (a) Radiography result shows Mrg15 has no histone methyltransferase activity. (b) Western blot results show the analysis of HMT reaction using antibodies against H3K36me1, H3K36me2 and H3K36me3. 300 ng (1000 ng for H3K36me1 analysis) core histones from Drosophila S2 cells were used as a control. (c) Radiography and scintillation results show that both hMrg15 and hMrgX stimulate the activity of Drosophila Ash1C. (d) Radiography and scintillation results show Mrg15Δchromo show less potency than wild-type Mrg15 in stimulation of Ash1C and Mrg15ΔMRG can not stimulate Ash1C. (e) Radiography and scintillation results Ash1C-Mrg15 complex has better activity than Ash1C-Mrg15Δchromo complex on titrated substrates. (f) Kinetic characterization of Ash1C and Ash1C-Mrg15 complex with titration of S-[methl-^{3}H] Adenosyl-methionine. Data are shown as mean ± s.e.m. (n=2). Note that due to limitation of S-[methl-^{3}H] Adenosyl-methionine concentration, it was not possible to reach saturation.

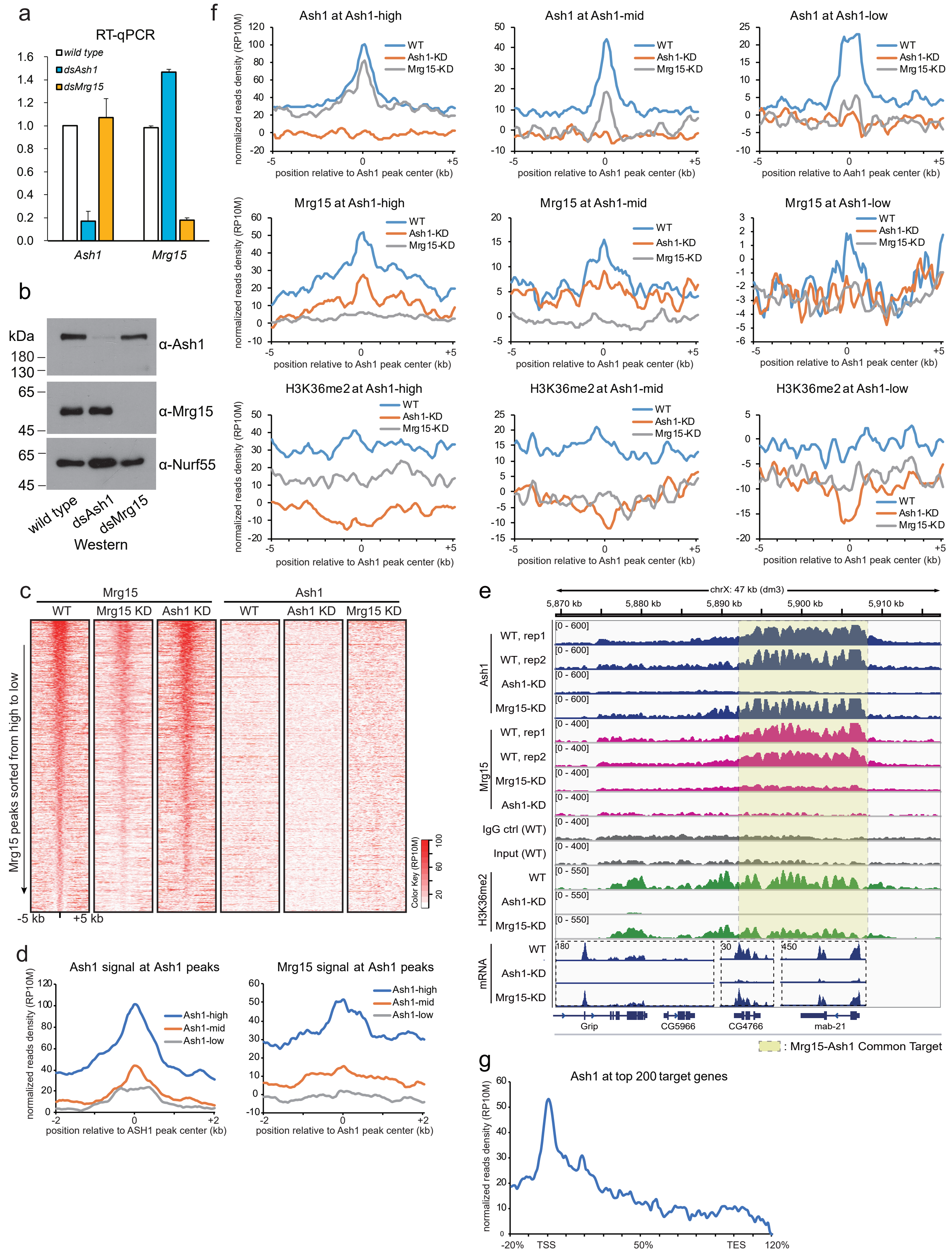

Supplementary Figure 3. Ash1 recruits Mrg15 to their common target genes and Mrg15 is required for proper deposition of Ash1-catalyzed H3K36me2 in S2 cells. (a) RT-qPCR result shows the efficiency of Ash1 and Mrg15 knockdown. Data are shown as mean + s.e.m. of two biological replicates (n=2). See also Supplementary Table 1 for RT-qPCR primers and Supplementary Table 2 for dsRNA targeting sequences. (b) Western result shows the efficiency of Ash1 and Mrg15 knockdown. (c) Heat maps show the occupancy of Mrg15 and Ash1 around Mrg15 peaks in wild-type, Mrg15 knockdown and Ash1 knockdown cells. Peaks were sorted according to Mrg15 reads densities in wild-type cells. (d) Profile of average normalized read densities (RP10M) show the occupancy of Ash1 and Mrg15 around three subgroup of Ash1 peaks (Ash1-high, Ash1-mid and Ash1-low). (e) Genome browser tracks show the binding of Ash1 and Mrg15, H3K36me2, and RNA-seq signals at several representative genes. (f) Profile of average normalized read densities (RP10M) show the occupancy of Ash1, Mrg15 and H3K36me2 around three subgroups of Ash1 peaks (Ash1-high, Ash1-mid and Ash1-low) in wild-type, Ash1 knockdown and Mrg15 knockdown cells. (g) Profile of average normalized read densities (RP10M) show the occupancy of Ash1 across the entire gene region of top 200 targets.

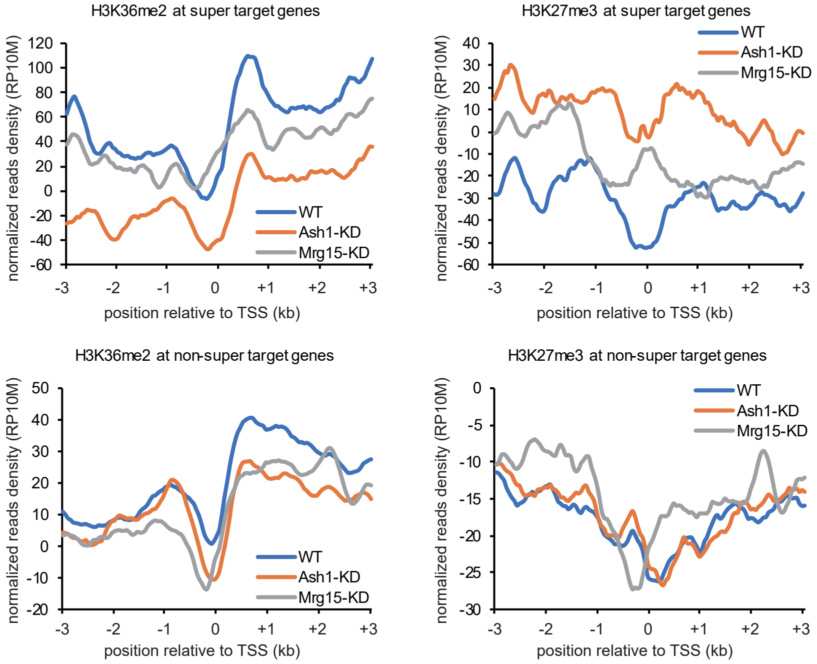

Supplementary Figure 4. H3K27me3 levels selectively increase at Ash1 super targets after Ash1 and Mrg15 depletion. Profile of average normalized read densities (RP10M) show the occupancy of H3K36me2 and H3K27me3 around TSS of Ash1 super target and non-super target genes in wild-type, Ash1 knockdown and Mrg15 knockdown cells.

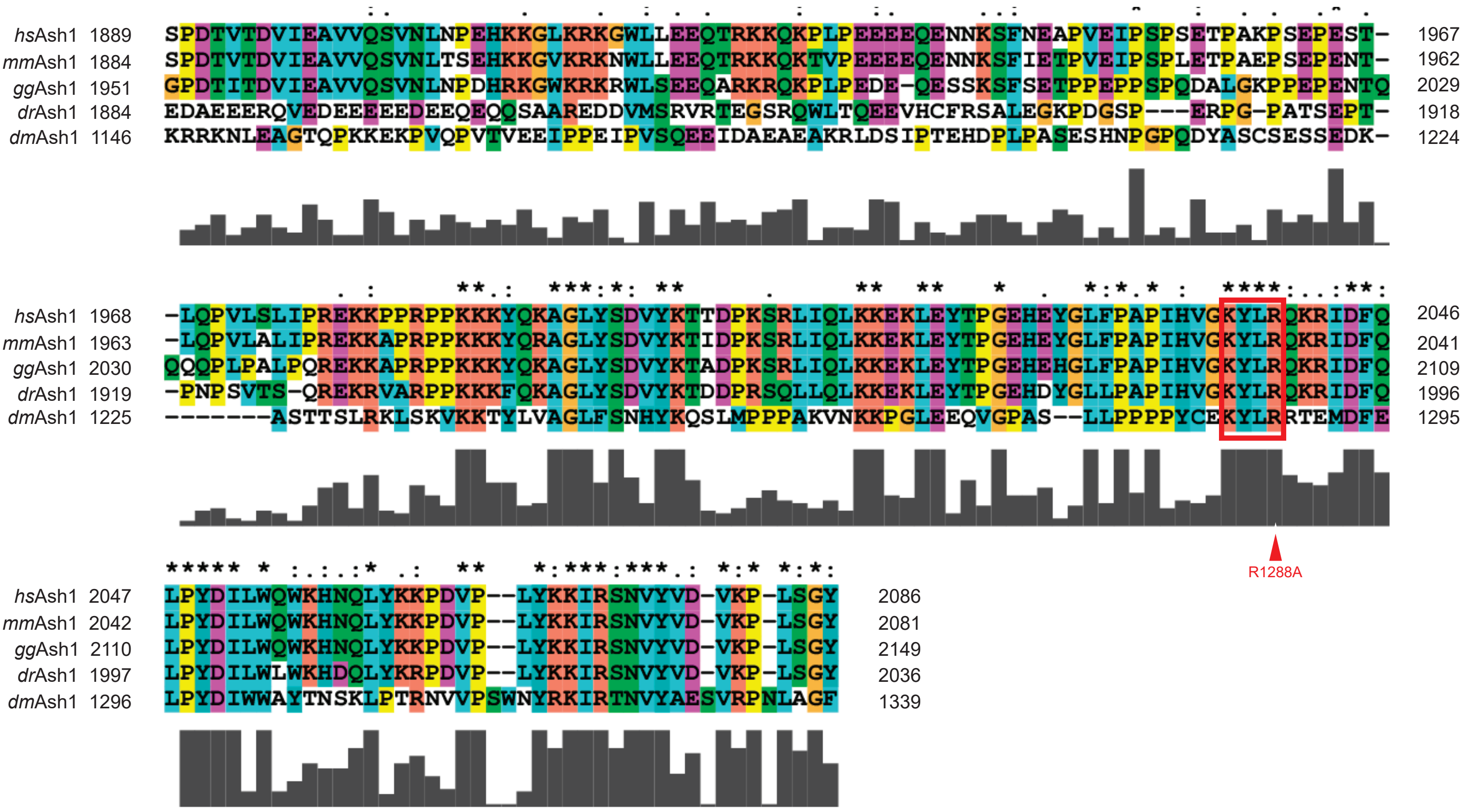

Supplementary Figure 5. Sequence alignment of Mrg15 interaction domain (MID) of Ash1 proteins from selected model organisms. The invariable KYLR motif was marked with a red box and R1288 in Drosophila Ash1 was marked by a triangle. Protein sequences used for the alignment are hsAsh1L (Q9NR48), msAsh1 (Q99MY8), ggAsh1 (XP_010722239), drAsh1 (NP_001333336) and dmAsh1 (Q9VW15).

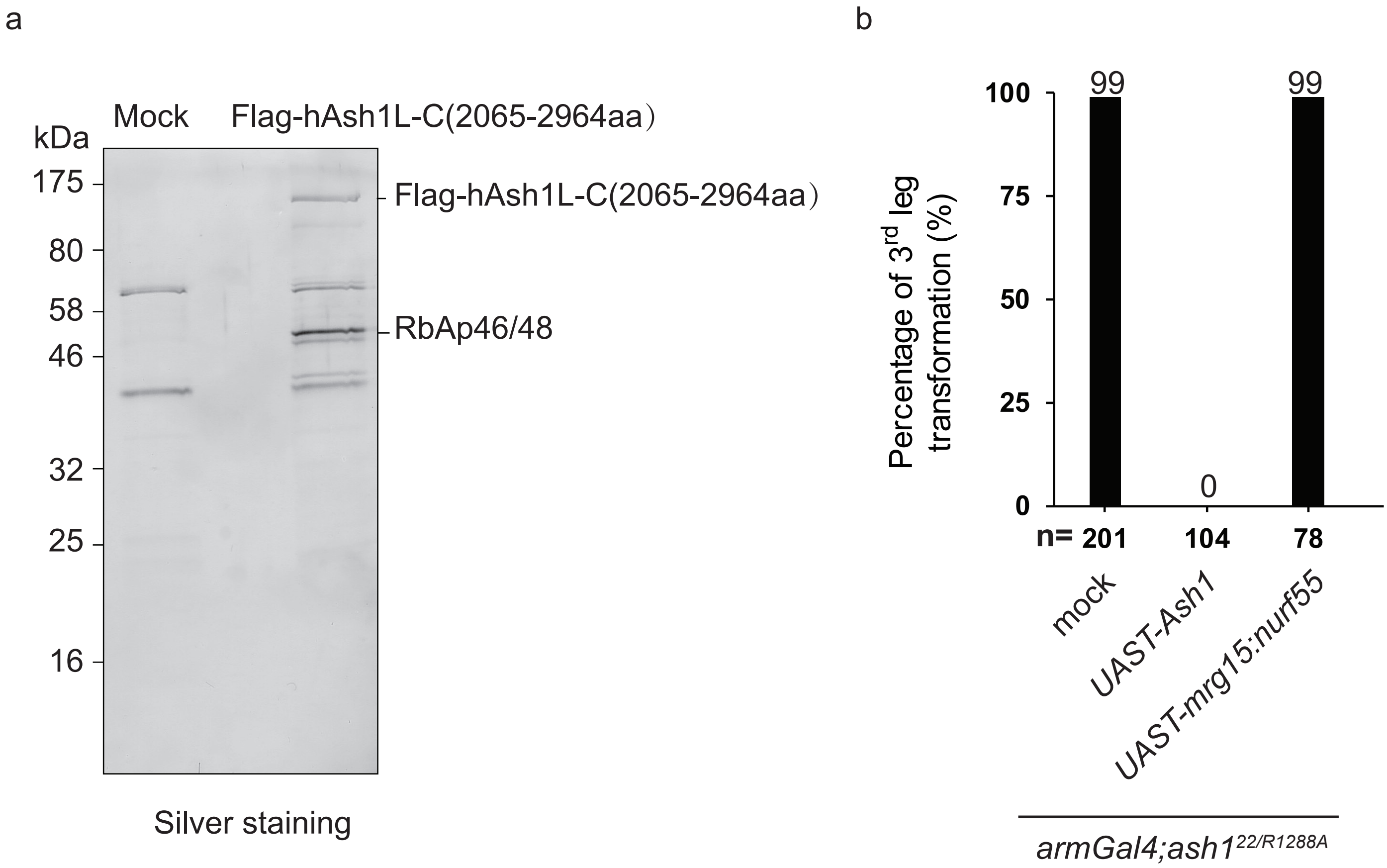

Supplementary Figure 6. Mrg15-Nurf55 fusion protein can not rescue the third leg to second leg transformation phenotype in ash122/R1288A fly. (a) Silver staining results show affinity purified materials from HEK293F cells expressing mock- or Flag-hAsh1L-C2 (2065-2964 aa). Note that only RbAp46/48 but not MORF4L1/2 copurified with this shorter form of hAsh1L C terminal fragment, indicating RbAp46/48 bind hAsh1L beyond the Mrg15 interaction domain. (b) Statistics data show the percentage of third leg to second leg partial transformation phenotype in flies of indicated genotypes. The exact sample size (n) for each genotype is indicated.

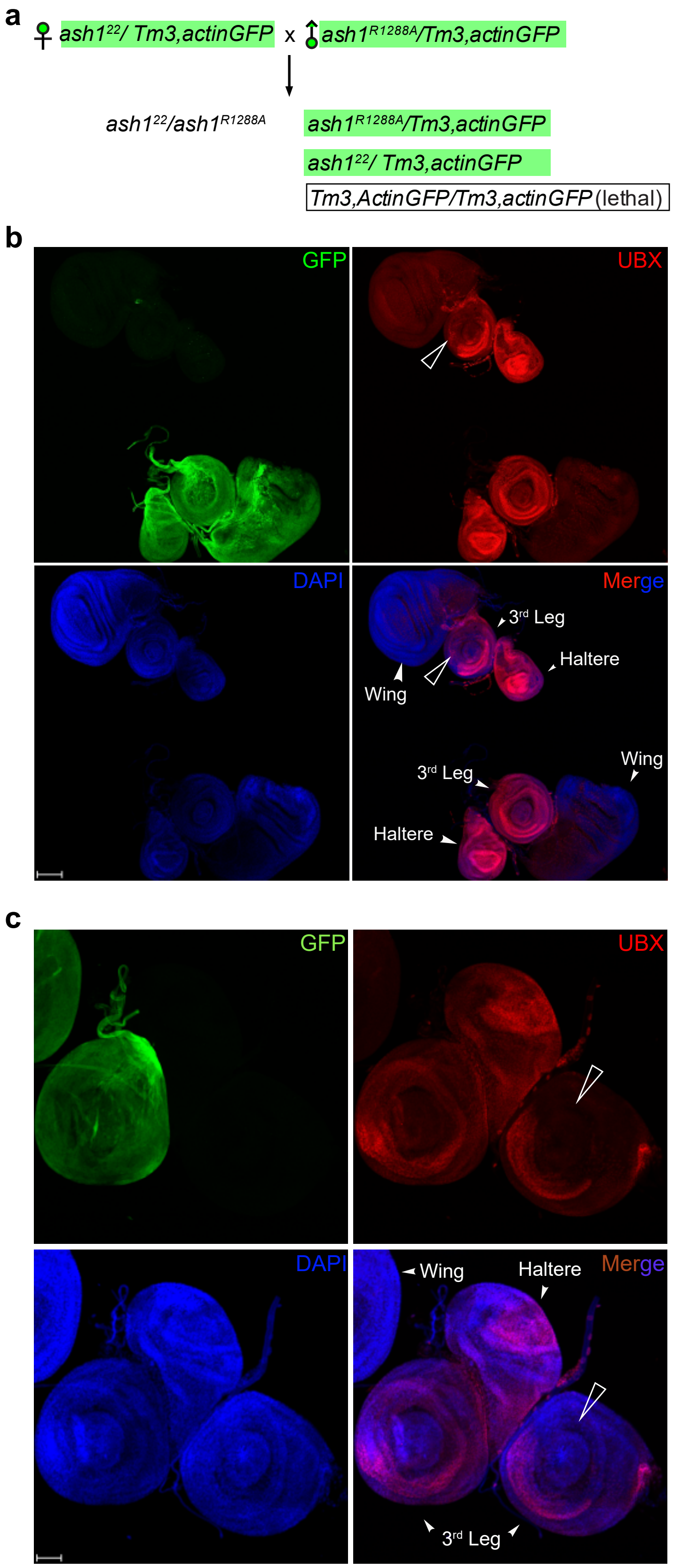

Supplementary Figure 7. R1288A mutation compromised UBX expression in 3rd leg imaginal discs. (a)Scheme of the cross. GFP positive discs contain one wild-type *ash1* allele, GFP negative discs are $ash1^{22/R1288A}$ mutants. (b, c) Immunostaining of UBX expression in imaginal discs. The defective regions are indicated with long arrowheads. Scale bars represent 100 µm (b) and 50 µm (c) respectively.

Fig. 1c uncropped western blots

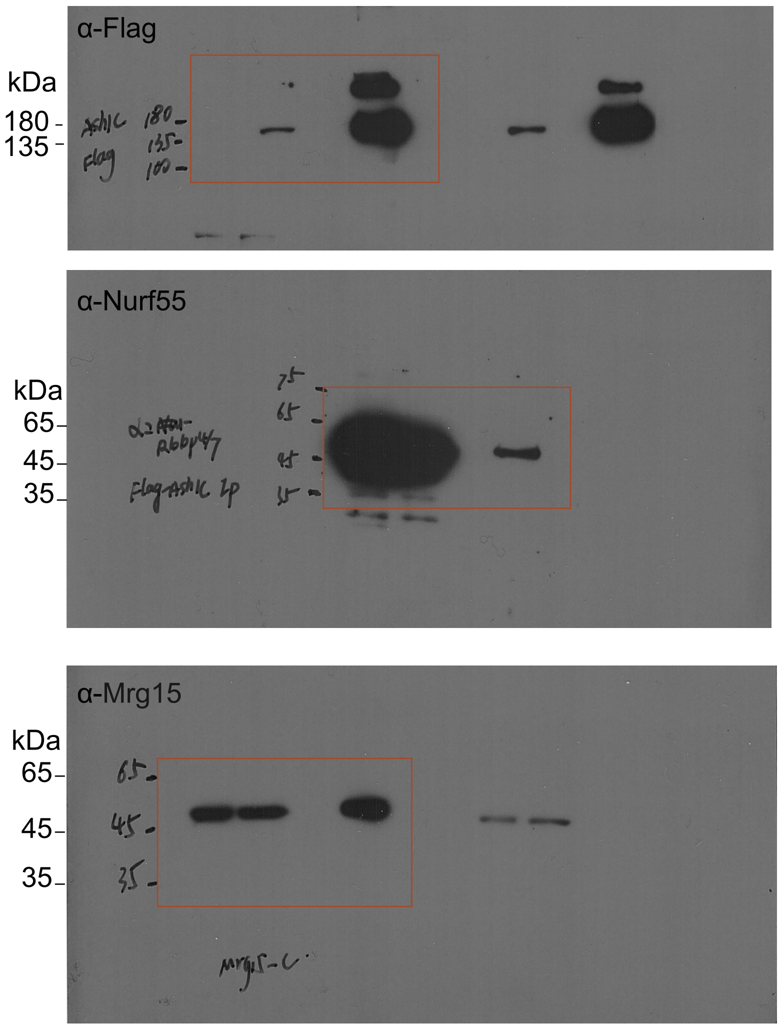

Fig. 1d uncropped western blots

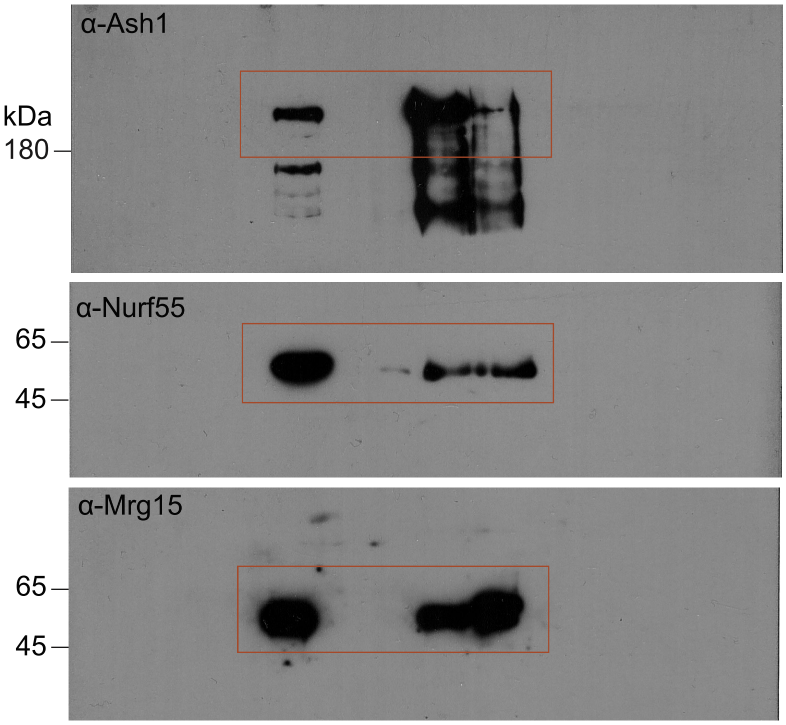

Fig.2a uncropped radiography result

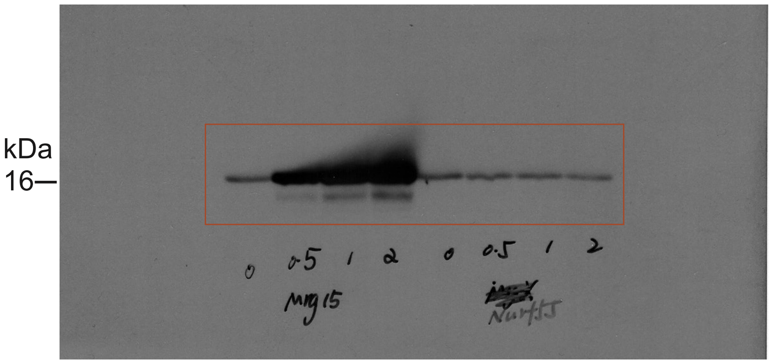

Fig.2b uncropped radiography result

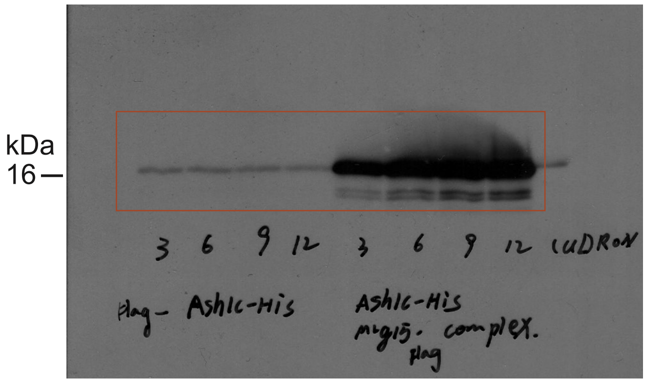

Fig.2c uncropped radiography result

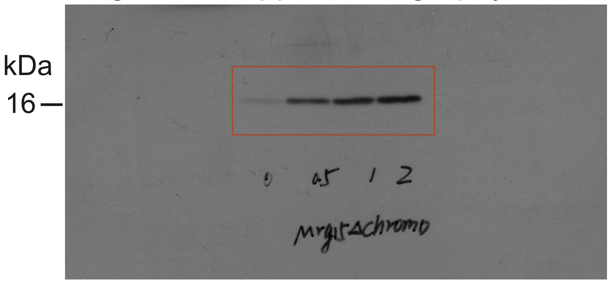

Supplementary Figure 8: Uncropped western blots and radiography images for Figure 1 and Figure 2.

Fig. 5b uncropped western blots

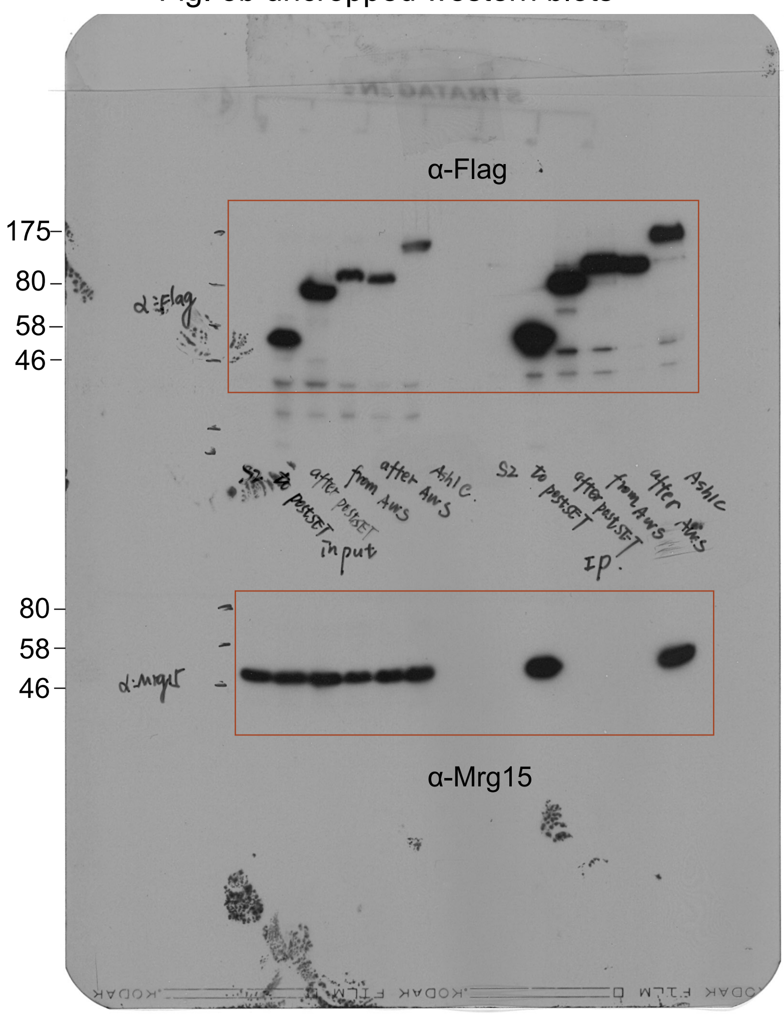

Fig. 5c uncropped western blots

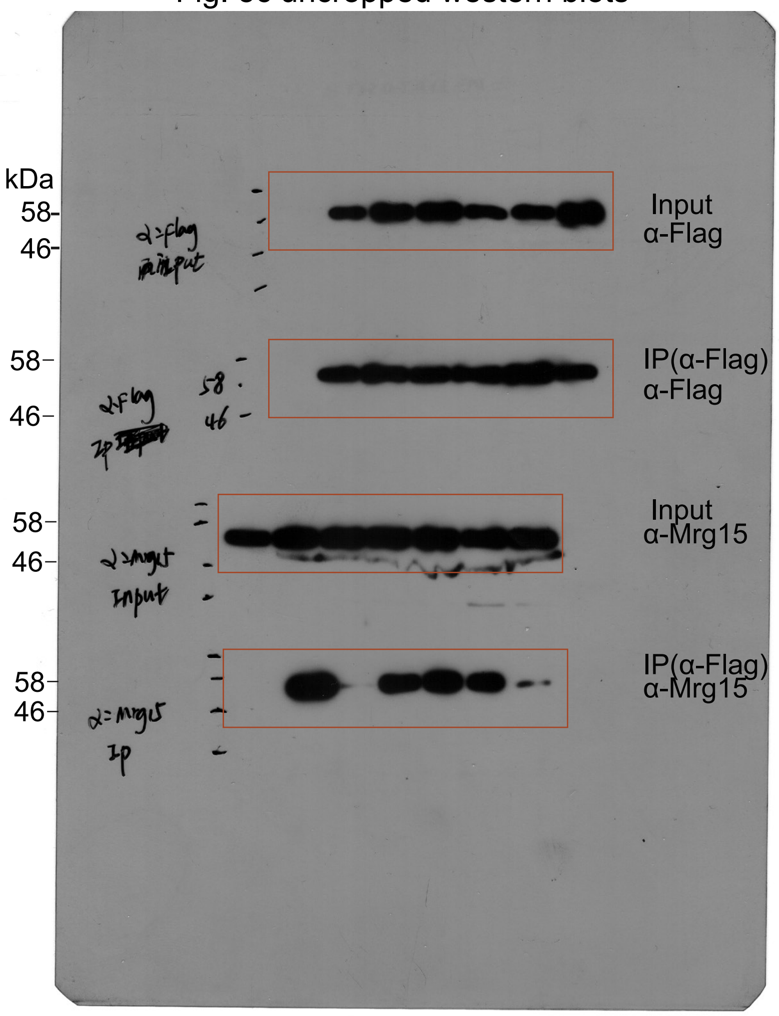

Fig. 5d uncropped radiography result

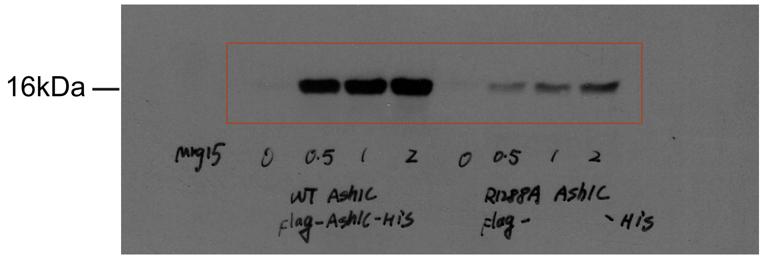

Supplementary Figure 9. Uncropped western blots and radiography images for Figure 5.

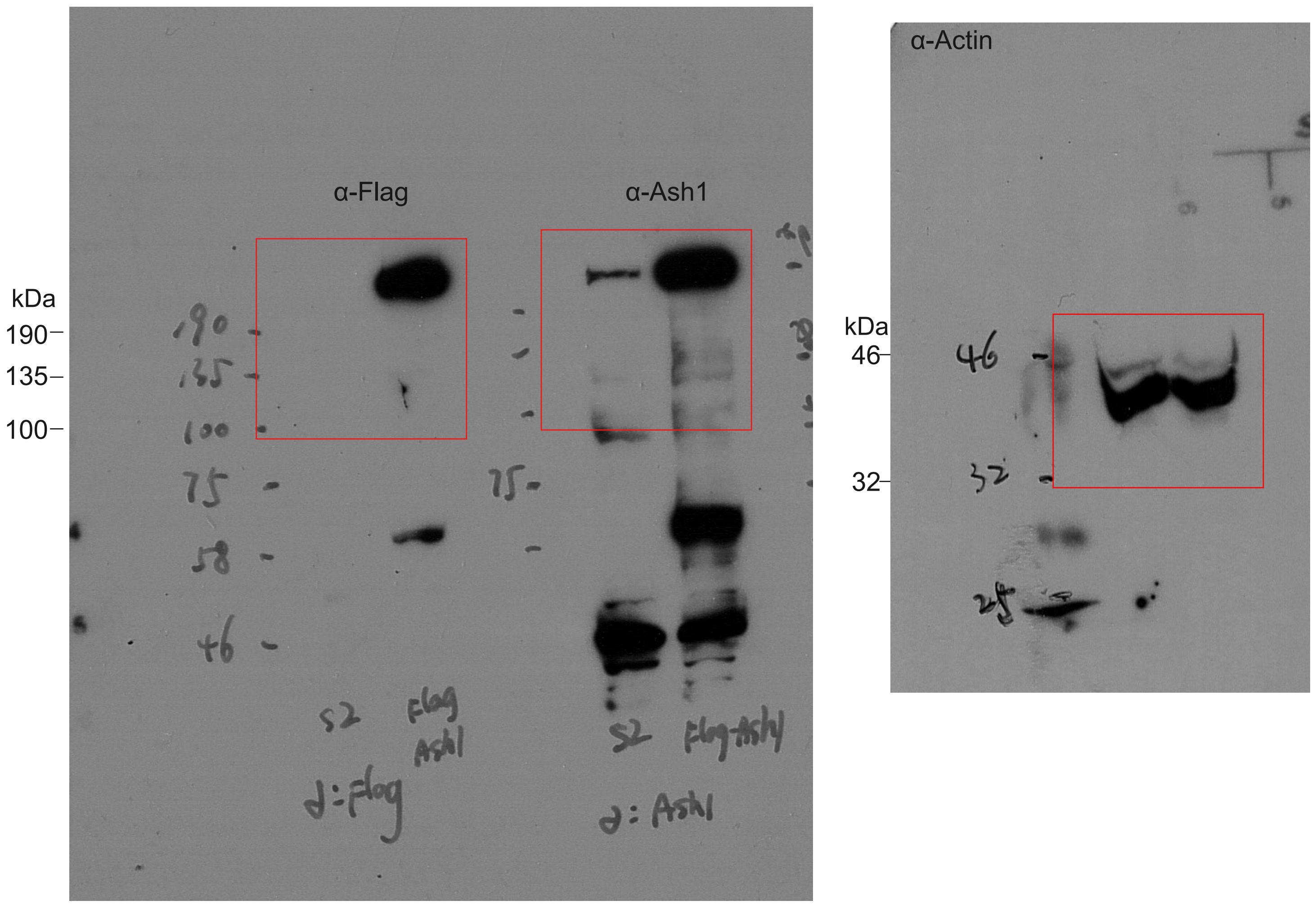

Supplementary Fig. 1d uncropped western blots

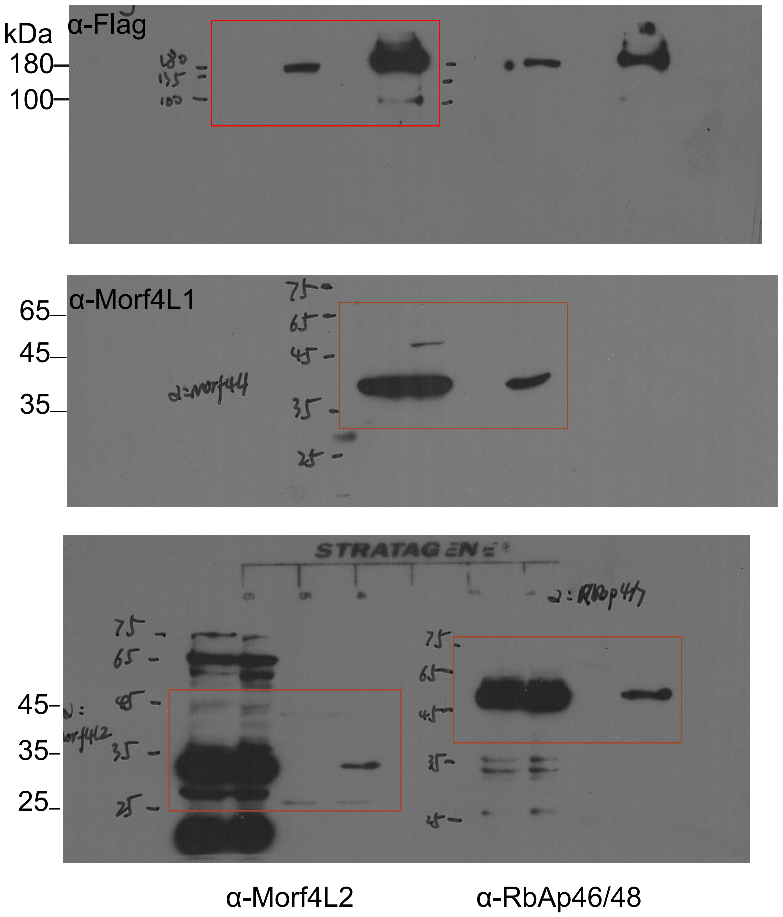

Suppmentary Fig. 1e uncropped western blots

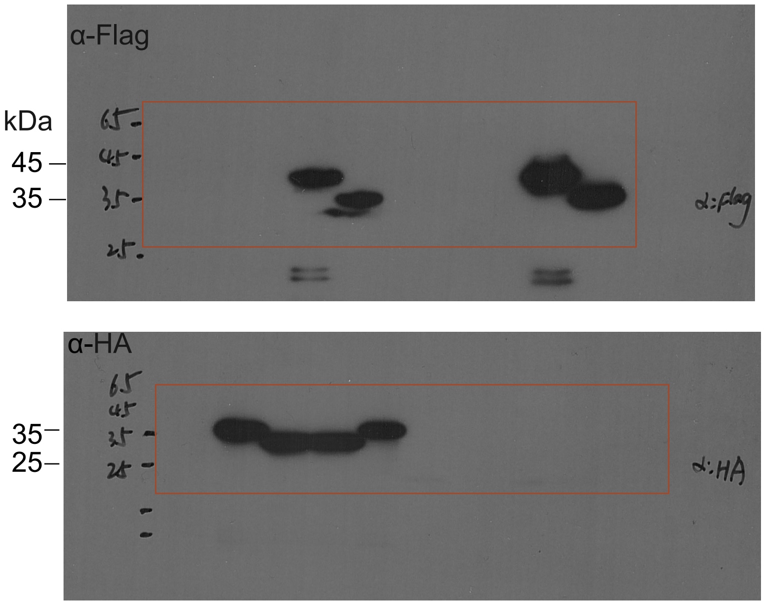

Supplementary Figure 10. Uncropped western blots images for Supplementary Figure 1.

Supplementary Fig. 2a uncropped radiography result

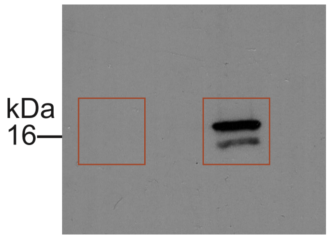

Supplementary Fig. 2b uncropped western blot

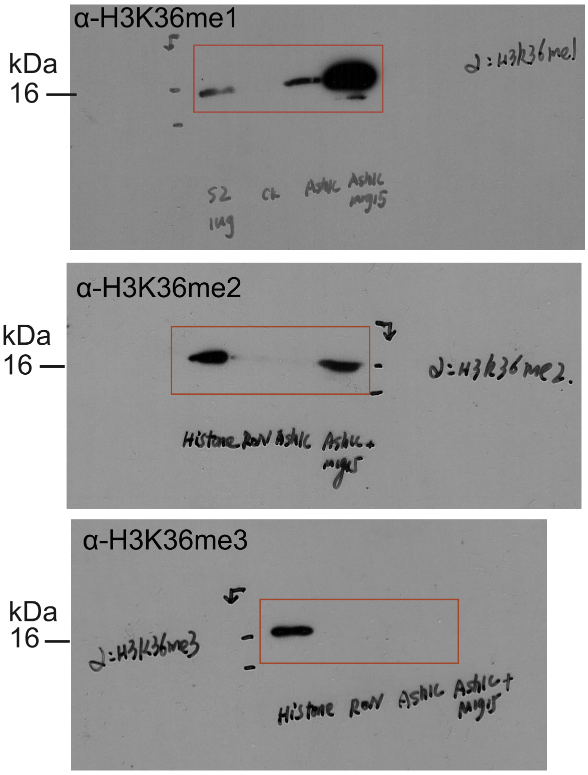

Supplementary Fig. 2c uncropped radiography result

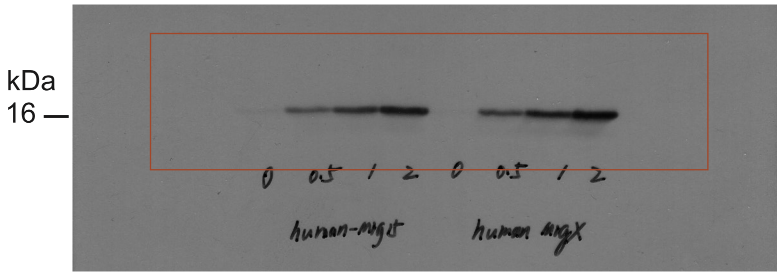

Supplementary Fig. 2d uncropped radiography result

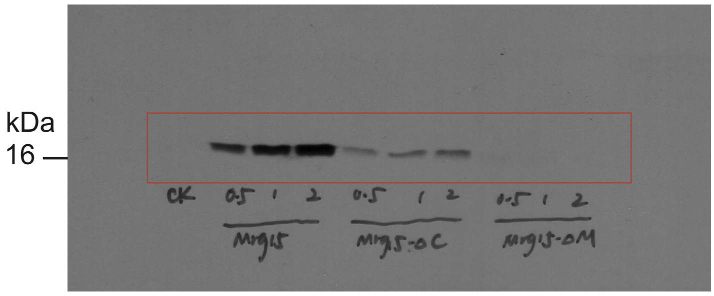

Supplementary Fig. 2e uncropped radiography result

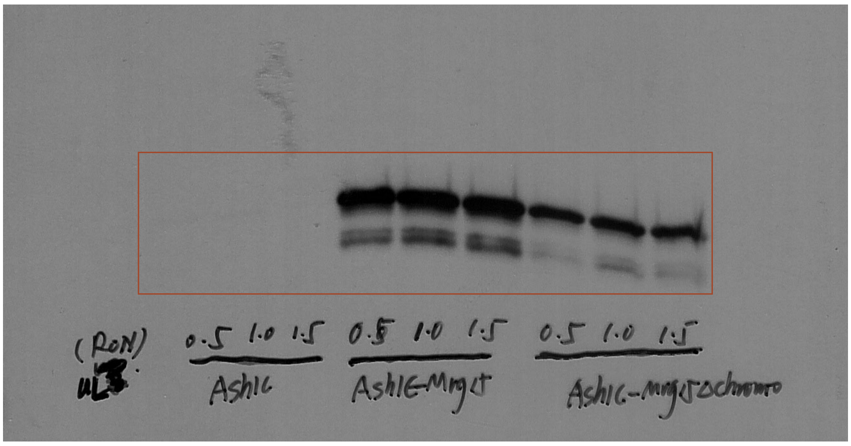

Supplementary Fig.3b uncropped western blots

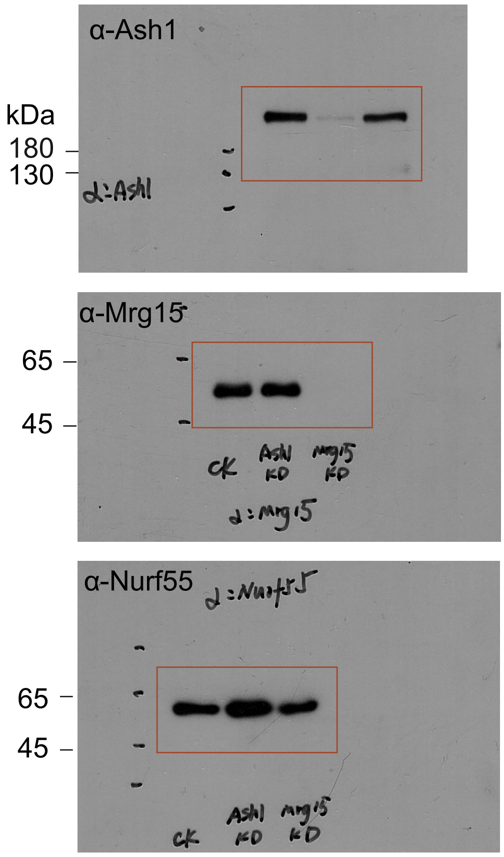

Supplementary Figure 11. Uncropped western blots and radiography images for Supplementary Figure 2 and Supplemmentary Figure 3.

Supplementary Table 1. RT-PCR primers.

Gene	Strand	Sequence(5' to 3')
ash1	F	AAGAGCTATGCGCCCCATG
ash1	R	ACGTTCTCTAAACGCGATTTC
mrg15	F	TGTTCGTGGATGGGGAAC
mrg15	R	CCACTCGTCCCAGTTTTTAC
gapdh	F	ATCGTCGAGGGTCTGATG
gapdh	R	ACGGTAAGATCCACAACG

Supplementary Table 2. The dsRNA targeting sequences.

dsRNA target	Sequence (5’ to 3’)
ash1	CTTTGTGGCCAGGACCAATCAAAAAGCCCCTCGATTATCGGTGGTG<br>GCCCTGGAGCGCCTACAGCGTCCTCAAACACCAGCTAGAGGAAGA<br>CCGCGAGGTAGAAAACCTAAGAACAGGGAACAAGCTGAAGCTGC<br>ACCTCAACCGCCGCCCAAATCGGAACCTGAGATAAGGCCAGCCAA<br>AAAACGTGGCCGGCAACCCAAGCAGCCGGTACTGGAAGAGCCAC<br>CACCCACACCACCTCCTCAACAGAAAAAAAACAAAATGGAACCAA<br>ATATTAGACTACCAGATGGCATCGATCCCAATACGAATTTCAGCTGC<br>AAGATTCGCTTGAAGCGGCGAAAGAACTTAGAGGCTGGAACCCAA<br>CCAAAAAAGGAGAAGCCAGTCCAGCCAGTGACGGTGGAAGAGAT<br>TCCACCAGAAATTCCCGTCAGTCAAGAAGAAATAGATGCAGAAGC<br>AGAGGCTAAACGGCTAGACAGTATTCCTACCGAGCACGATCCCTTG<br>CCTGC
mrg15	AATCGACCAGTGCCAGCAAGGAGGTTGCCATAAACGATGTACTCG<br>ACGGAATTGGAGAGTACTTCAATGTAATGCTGGGCTCCCAGTTGCT<br>GTACAAATTCGAACGCACCCAGTACGCGGATGTGATGCAGAAGCAT<br>CCGGACACACCGTTGTCCGAGCTTTACGGATCTTTTCACCTGCTGC<br>GTCTGTTTGTCCGCCTTGGCTCAATGCTCAGCTACTCTGCGTTGGAT<br>CAGCAGTCCATGCAGAACCTACTCACGCACGTGCAGGATTTCCTTA<br>AATTCCTCGTAAAGAACAGCTCAATATTTTTCAGCATGAGCAACTTT<br>ATCAACGTTGATCCCGAGTACGTGCGAAATGCACAGTAAGCAATAA<br>TCTTTTCTTGACTTGAAGTAAATATTTCATCAAAACAGTTTGCAATA<br>ATTCGGTGGTTGCTCTCAATTTGCTCCCAAA
