# Supplementary material for: Mrg15 stimulates Ash1 H3K36 methyltransferase activity and facilitates Ash1 Trithorax group protein function in Drosophila
Source: Nat Commun. 2017 Nov 21;8:1649. doi: 10.1038/s41467-017-01897-3 (PMC5696344; doi:10.1038/s41467-017-01897-3)
Supplement: Supplementary file 3 — Description of Additional Supplementary Files [file 41467_2017_1897_MOESM3_ESM.pdf]

Description of Additional Supplementary Files

File Name: Supplementary Data 1
Description: List of all Ash1 target genes and their expression levels in wild-type, Ash1 knockdown and Mrg15 knockdown S2 cells.
